# The Use of Absorbable Plates to Treat Facial Fractures From Dog-Bite Injuries in Pediatric Patients: A Case Report

**DOI:** 10.7759/cureus.66532

**Published:** 2024-08-09

**Authors:** Alessandra Manzali Flores, Julio R Castillo-Moreno, Miguel E Viera-Nuñez, Alfredo A Carballo-Magdaleno, Danae Tapia-Alquicira

**Affiliations:** 1 Plastic Surgery, Hospital General Dr. Rubén Leñero, Mexico City, MEX; 2 Pediatric Plastic Surgery, Hospital Pediátrico de Tacubaya, Mexico City, MEX; 3 Plastic Surgery Residency, Hospital General Dr. Rubén Leñero, Mexico City, MEX; 4 Orthodontics and Dentofacial Orthopedics, Hospital Pediátrico de Tacubaya, Mexico City, MEX

**Keywords:** dog bites wound, absorbable material, pediatric facial fractures, facial fractures, dog-bite

## Abstract

Dog-bite injuries are common in the facial and neck areas of pediatric patients on account of their size. The incidence of dog-bite etiology for facial fractures in Mexico is unknown as they are underreported. We present a case of a pediatric patient with facial fractures due to dog-bite injuries. We describe the patient's surgical management with absorbable plates and its aftermath and engage in a literature review of dog-bite facial fractures. The patient demonstrated generally favorable results, with minimal postsurgical sequelae. The use of absorbable plates leads to positive outcomes in pediatric patients with dog-bite-related facial fractures.

## Introduction

Dog-bite-related injuries are commonly encountered in the facial and neck areas of pediatric patients because of their size. The incidence of dog-bite etiology for facial fractures in Mexico is currently not documented owing to their underreporting. Also, there is scarce data on the incidence of dog-bite facial fractures in pediatric patients, with only 41 reported cases in the literature until 2018, as per the study by Heitz et al. [1]. Facial fractures in children are associated with high morbidity, and an interdisciplinary approach is required to treat them [2]. In the study by Tu et al., 5% of dog bites in the head and neck manifest facial fractures [3]. However, 50% of cases of facial fractures involve multiple fractures, and hence the discovery of one fracture must prompt the exploration of a second fracture [4].

## Case presentation

**Figure 1:**
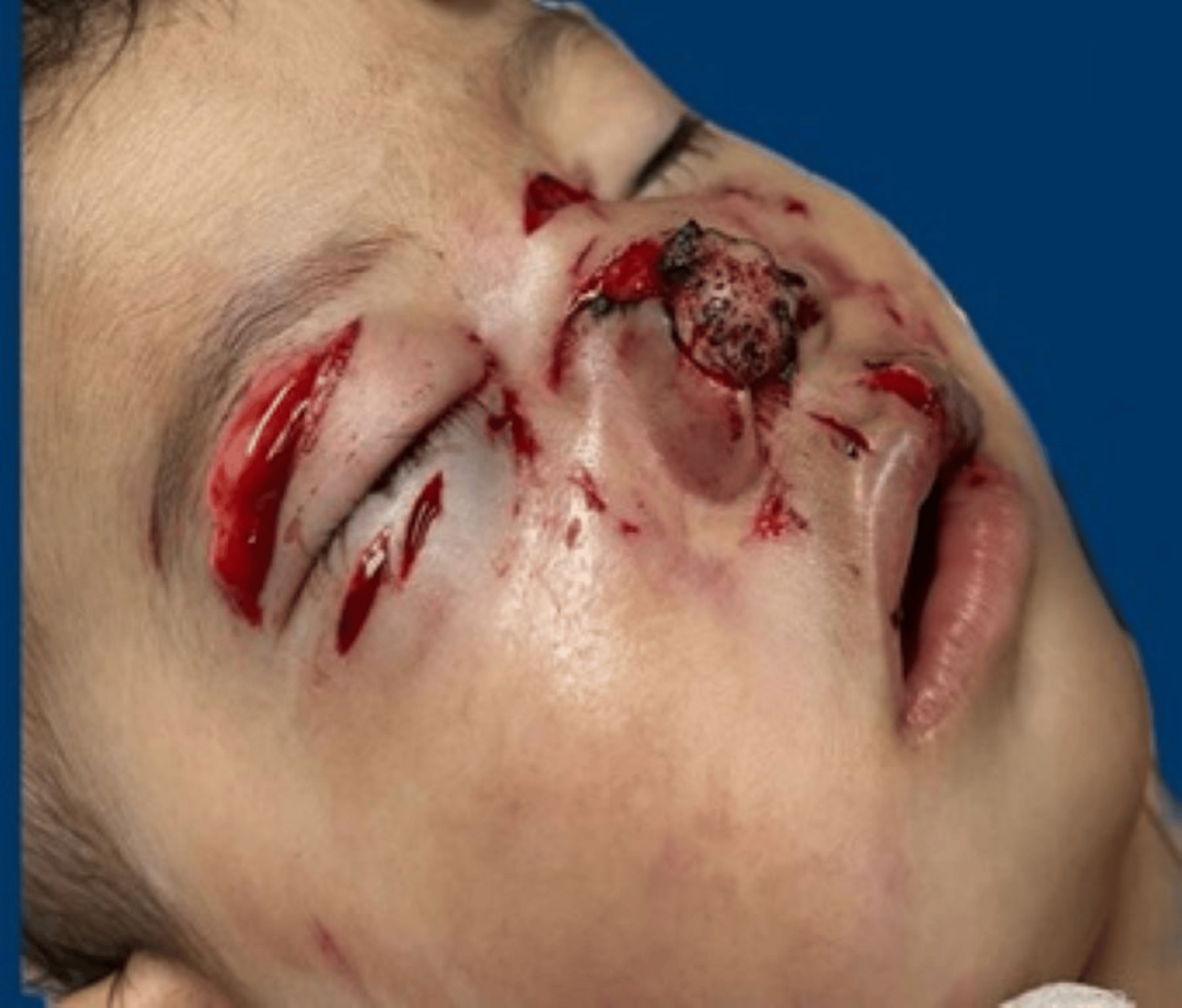
Presurgical image

An 11-month-old female patient presented to Tacubaya Pediatric Hospital's plastic surgery department with dog bites in the facial area on November 26, 2023. Upon arrival, the patient had multiple facial injuries, a superior eyelid with edema, a linear wound without active bleeding, and two 1 cm linear wounds on the inferior eyelid without active bleeding or tissue loss. Also, multiple irregular wounds in the nasal tip, a 1 cm wound in the filtrate and upper left lip, and crepitation and tenderness in the right malar bone were observed, along with osseous fragments evident through the left superior eyelid wound (Figure 1). There was also an associated conjunctival wound with orbital fat protrusion in the right eye, which required ophthalmologic assessment and treatment.

**Figure 2:**
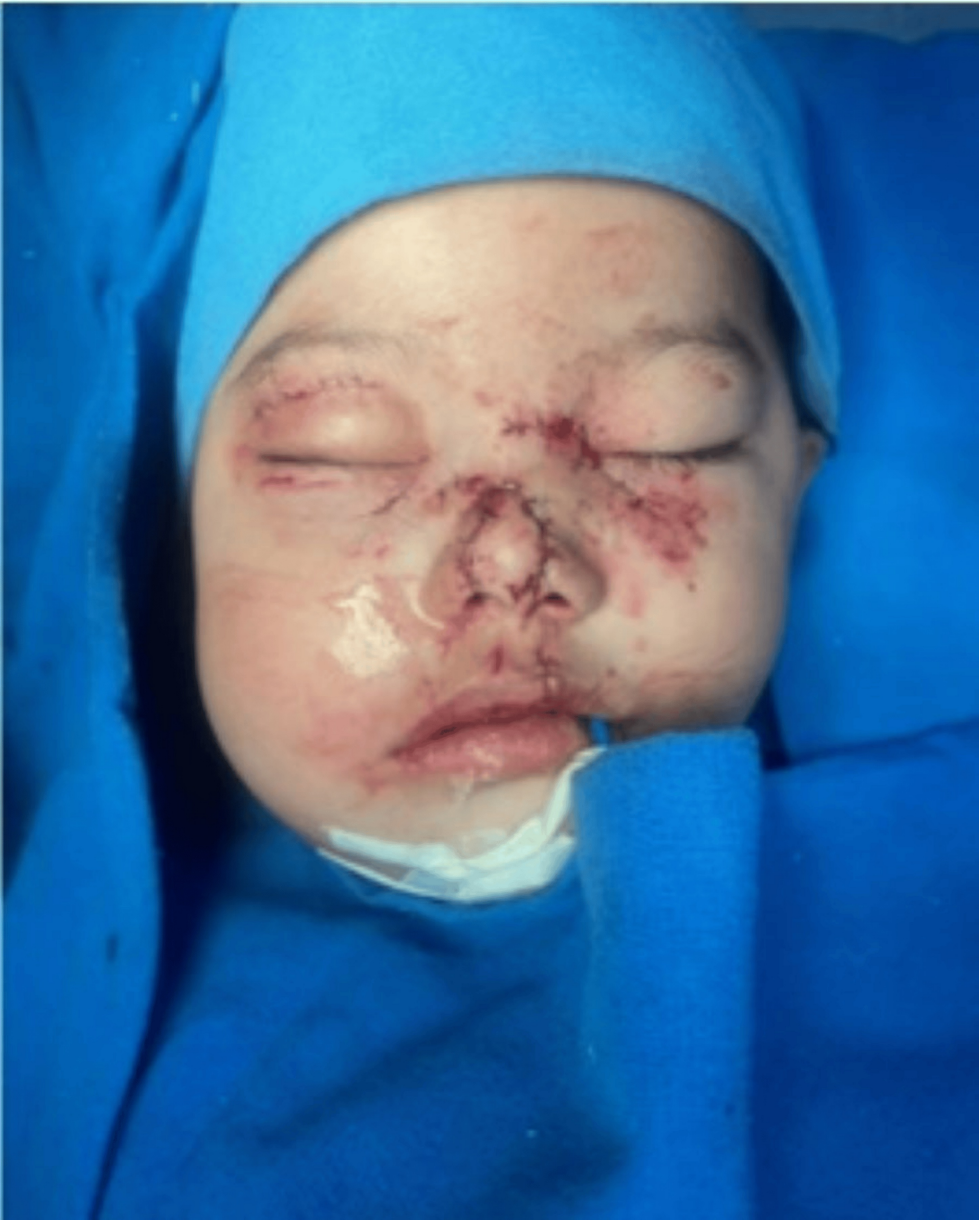
Immediate postoperative image after first surgery

**Figure 3:**
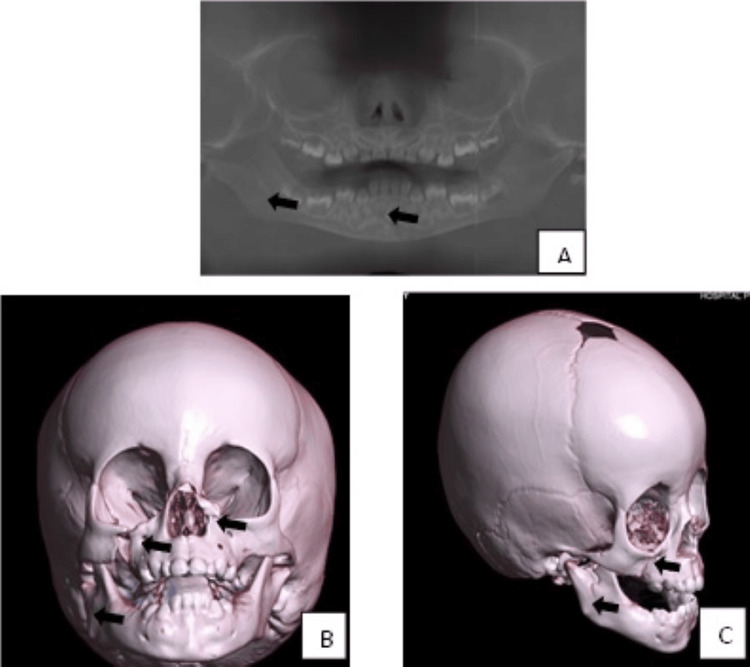
Presurgical orthopantomography and tomographic 3D reconstruction A: Orthopantomography; unicortical symphyseal fracture and high ramus mandibular condylar fracture. B and C: Simple 3D CT reconstruction; condylar fracture Kohler V, orbitomalar right Knight North V fracture, right orbital fracture, unicortical symphyseal fracture, and nasal fracture CT: computed tomography

The surgical procedure was performed after obtaining parental consent on November 29, 2023, for wound closure (Figure 2). Postsurgical surveillance and intravenous antibiotics were administered. A tomography study was performed to identify the right condylar Kohler V, right orbitomalar Knight North V, right floor orbital, unicortical mandibular symphysis, and nasal fractures (Figure 3). An additional surgical procedure was scheduled for December 4, 2023.

Internal fixation was performed with BioSorb material, using a transparotid approach (through a previous wound). A complex condylar fracture was found, with associated bone deformity due to a green stem-associated fracture. The vestibular and transconjunctival approach was completed by placing a three-hole absorbable plate, for malar reduction, and malar-maxilar buttress fixation. We used Histoacryl (tissue glue) for nasal bone reduction.

**Figure 4:**
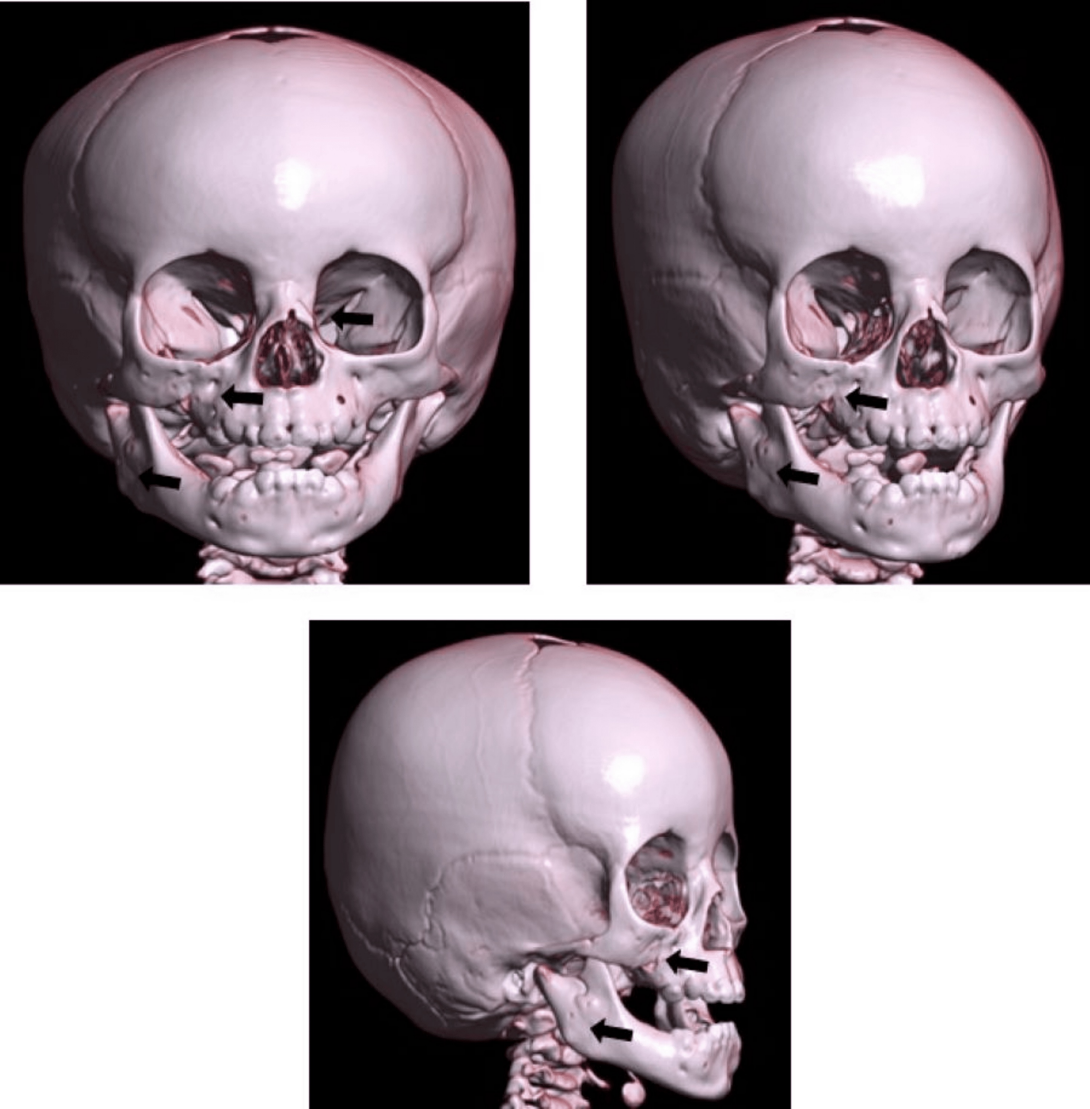
Tomographic reconstruction: postsurgical images Simple 3D CT reconstruction; reduced condylar Kohler V, orbitomalar right Knight North V, right orbital fracture, unicortical symphyseal, and nasal fractures with absorbable polylactic acid plates CT: computed tomography

A postsurgical CT was taken (Figure 4), which showed adequate fracture reduction. Clinically presenting right eyelid ptosis was observed, without facial nerve damage. Two months after the surgery, the patient presented with persisting right eye-lid elevator damage, no eye movement, and limitations in mouth movement. Four months after the procedure, the patient exhibited improvement in palpebral elevation but she continues to have issues with eye and mouth movement.

## Discussion

The power of a dog's bites is proportional to the size of its mandible and muscles. The most common breeds implicated in fractures in humans are Pitbull, German Shepard, and Rottweiler [1,4,3,5]. Studies have reported that dog bites cause malar, nasal, and orbital fractures, associated with soft tissue damage of the nose and lips [1,5,6]. Children have 4.2 times more probability of presenting with eye lesions, as well as facial trauma, compared with adults [7] and more damage to the head and neck, due to their small stature and size of their head [3,8]. Most of the infections associated with dog bites are polybacterial. Pasteurella is the most isolated pathogen, observed in 50% of dog bites [3,5]. The gold standard antibiotic management for dog bites is amoxicillin with clavulanate acid, and it is very effective against aerobic and anaerobic bacteria; it is administered for 10-14 days if the wound is vast or has bone involvement [1,3 9,10,11]. However, antibiotic prophylactic treatment is also important, for dog bites are associated with an infection rate of 0-12% without antibiotic treatment [4].

Facial fractures in pediatric patients absorb more energy during the impact due to a greater proportion of cancellous bone and cartilaginous structures [1]. The patient's dentition status has implications for lesion patterns, as well as strategies for trauma management [12]. The localization of fractures is modified from superior to inferior in relation to the face; as age increases, this is secondary to mandibular and malar growth, with a progressive decrease of elasticity after two to three years of age [7]. Severe dog-bite face injuries must be evaluated with CT, in addition to the soft tissue evaluation [13]. Facial fracture reduction is key to a favorable postsurgical result, and it is associated with more difficulty in mandibular fractures, due to its force and constant motility [14]. The use of absorbable material, with polylactic and polyglycolic acid, has been recommended to prevent growth restriction and avoid the need for reintervention for the withdrawal of the titanium material [15]. Absorbable osteosynthesis material is weaker than its metallic counterparts but has fewer corrosive properties [16].

The fundamental attributes of fixation materials in facial fractures are biocompatibility, strength, ductility, and adaptability to the bone´s surface. The absorbable material offers greater benefits by avoiding migration and interference in bone growth as it expands [17] According to a meta-analysis by Chocron et al., absorbable material for mandibular fractures is a viable alternative to metallic osteosynthesis, with similar rates of complication and functional results [18]. With the emergence of new technologies in materials for bone repair, the number of pediatric patients treated with open reduction and internal fixation has increased. However, several disadvantages have been highlighted, such as higher surgical time for heat plate molding, insufficient resistance for mandibular fractures, and foreign body reaction [19]. Studies report reabsorption in 12 months, but some cases requiring longer periods (up to 5 years) [17] have also been described, which raises concerns regarding intense inflammatory reactions.

## Conclusions

While dog bite-associated facial fractures are common, they are underreported in the literature. They often require surgical and multidisciplinary treatment to avoid complications. In our case, an adequate functional result was attained with the use of absorbable plates. Further studies are required to gain deeper insights into the use of this technique in pediatric patients.
